# A Case of Malan Syndrome With Pulmonary Artery Dilatation due to a Novel Frameshift Variant in Exon 2 of the *NFIX* Gene

**DOI:** 10.1155/crig/4265185

**Published:** 2026-06-16

**Authors:** Toshihiko Mori, Mayu Hirano, Shigeto Fuse, Maki Katai, Hiroko Shigetomi

**Affiliations:** ^1^ Department of Pediatrics, NTT Medical Center Sapporo, Sapporo, Hokkaido, Japan; ^2^ Department of Laboratory, NTT Medical Center Sapporo, Sapporo, Hokkaido, Japan; ^3^ Department of Ophthalmology, NTT Medical Center Sapporo, Sapporo, Hokkaido, Japan; ^4^ Department of Pediatrics, JCHO Sapporo Hokushin Hospital, Sapporo, Hokkaido, Japan

## Abstract

Malan syndrome is a rare overgrowth disorder caused by deletions or pathogenic variants in the *NFIX* gene. Here, we present the case of a Japanese male infant with Malan syndrome, which was caused by a novel frameshift variant resulting from an eight‐base insertion in Exon 2 of the *NFIX* gene. Echocardiography revealed dilatation of the pulmonary artery without hemodynamic abnormalities. Malan syndrome shares many clinical features with Sotos syndrome, including overgrowth, macrocephaly, developmental delay, and intellectual disability. Therefore, molecular genetic testing is required for diagnosis. Pathogenic variants in the *NFIX* gene have also been reported in individuals with Marshall–Smith syndrome. Some individuals exhibit phenotypic overlap between Malan syndrome and Marshall–Smith syndrome. Our patient was diagnosed with Malan syndrome because he had a frameshift variant in Exon 2, resulting in haploinsufficiency, and exhibited macrocephaly, but not proptosis or micrognathia. To our knowledge, this is the second reported case in the literature of concomitant pulmonary artery dilatation in Malan syndrome.

## 1. Introduction

Malan syndrome (MIM #614753) is a rare overgrowth syndrome formerly diagnosed as Sotos Syndrome 2 or Sotos‐like syndrome. It is characterized by overgrowth, macrocephaly, developmental delay, and intellectual disability. The syndrome is caused by deletions or pathogenic variants in the *NFIX* gene [1, 2]. Malan syndrome is an autosomal dominant disorder; however, nearly all reported cases involve de novo variants. Malan syndrome is also characterized by symptoms reminiscent of Marfan syndrome, such as thin body habitus, long fingers, scoliosis, and chest deformities (pectus carinatum/excavatum), which are caused by abnormalities in the TGF‐β pathway. These symptoms raise concerns about possible complications, such as aortic root dilatation. However, only one case of pulmonary artery dilatation and five cases of aortic dilatation have been reported in previous studies of cardiovascular disease in Malan syndrome [2–5]. Here, we report on a Japanese male infant with Malan syndrome and pulmonary artery dilatation due to a novel frameshift variant in Exon 2 of the *NFIX* gene.

## 2. Case Report

**FIGURE 1:**
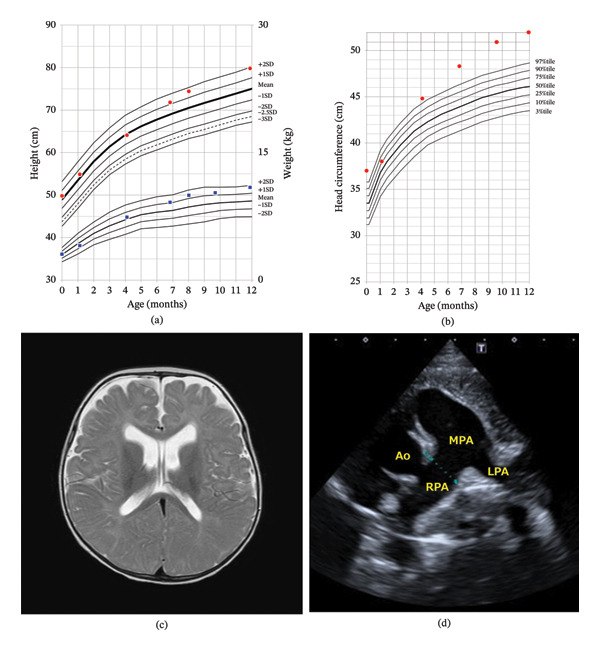
(a) Growth chart of the patient. Closed red circles indicate height, and closed blue squares indicate weight. (b) Head circumference‐for‐age percentiles for boys (birth to 12 months) from the normal Japanese growth standards. The patient’s head circumference at timepoints from birth to 12 months of age is plotted (in red, closed circles) against the normal Japanese growth percentiles. (c) Plain head magnetic resonance imaging (MRI). The head MRI (T2‐weighted image) showed mild ventricular enlargement. (d) Transthoracic echocardiography. Transthoracic echocardiography revealed pulmonary artery dilation (pulmonary artery root diameter > aortic root diameter). Ao: aorta; MPA: main pulmonary artery; RPA: right pulmonary artery; LPA: left pulmonary artery.

The patient was the first child of two healthy, unrelated Japanese parents. He was born via C‐section at 38 weeks and 2 days of gestation without asphyxia. His birth weight, height, and head circumference were 3054 g (−0.37 standard deviation [SD] from the mean for normal Japanese boys), 49.8 cm (+0.11 SD), and 37 cm (+2.5 SD), respectively. At 6 months of age, he was referred to our hospital for evaluation of an enlarged head circumference and developmental delay. Figure 1a and b show the results of the physical measurements, which revealed a head circumference of 48 cm (+3.75 SD). His muscle tone was good, but he was unable to hold his head up or roll over in bed. The patient was shown at 8 months of age, exhibiting macrocephaly, a long triangular face, frontal bossing, down‐slanting palpebral fissures, and a pointed chin. AI facial recognition software (Face2Gene) identified Sotos syndrome as the primary candidate, with Malan syndrome as the secondary candidate. Head magnetic resonance imaging (MRI) revealed mild ventricular enlargement (Figure 1c). Chromosomal tests, such as G‐banding and fluorescence in situ hybridization (FISH) of Chromosome 5, revealed no abnormalities. Figure 1a and b show the patient’s physical measurements at 8 months of age. At that time, he could hold up his head and roll over in bed. An ophthalmologic examination revealed bilateral optic nerve atrophy and intermittent exotropia. Echocardiography revealed pulmonary artery dilatation (main pulmonary artery diameter > aortic root diameter), but no right ventricular hypertrophy or tricuspid regurgitation (Figure 1d). Spinal X‐ray revealed no scoliosis. Electroencephalography revealed no epileptic discharges.

We performed molecular genetic panel testing using next‐generation sequencing (NGS), targeting the *NSD1* and *NFIX* genes, to analyze the exons and exon–intron junction regions. The *NSD1* gene is responsible for Sotos syndrome, while the *NFIX* gene was used for differential diagnosis. No pathogenic variant was detected in the *NSD1* gene, but a novel heterozygous frameshift variant (NM_001271043.2: c.290_297dup) was detected in the *NFIX* gene. This variant causes a frameshift due to the insertion of eight bases, resulting in an early stop codon. This null variant is classified as “Pathogenic” according to the criteria of the ACMG guidelines (PVS1 +PM2+ PP4). Although a segregation analysis could not be performed, neither parent showed any clinical features suggestive of Malan syndrome.

## 3. Discussion

Malan syndrome and Sotos syndrome [6] share many clinical similarities, including overgrowth, macrocephaly, developmental delay, and intellectual disability. These similarities make it difficult to differentiate between the two based on clinical symptoms alone (Table 1). Molecular genetic testing is required for diagnosis. The early truncating variant detected in this case is predicted to undergo nonsense‐mediated decay, leading to NFIX loss‐of‐function, which is a known disease mechanism (haploinsufficiency) in Malan syndrome. Since most cases of Malan syndrome involve de novo variants, we did not screen the parents of this patient for variants. Therefore, we cannot definitively conclude that this case involves a de novo variant. Scoliosis, autism spectrum disorder, anxiety, and noise sensitivity are common in adults with Malan syndrome but are not apparent in infancy. Findings such as macrocephaly, overgrowth, developmental delay, and a protruding forehead cannot distinguish Malan syndrome from Sotos syndrome [2]. In this case, Sotos syndrome was the first candidate identified by the AI facial recognition software (Face2Gene). Ophthalmological abnormalities, such as optic atrophy and exotropia, are relatively rare in Sotos syndrome (Table 1) [2].

**TABLE 1:** Comparison of characteristics of Malan syndrome to those of Marshall–Smith syndrome and Sotos syndrome.

	Malan syndrome	Marshall–Smith syndrome	Sotos syndrome
Gene	*NFIX*	*NFIX*	*NSD1*
Common exons for deletions or variants	Clustered in Exon 2	Scattered through exons 6 to 10	
Causative mechanism	Haploinsufficiency	Dominant‐negative	
Prevalence	1/1,000,000	≤ 1,000,000	1/14,000
Growth			
Macrocephaly	+++	−	+++
Postnatal overgrowth (postnatal height ≥ 2SDS)	++	−	+++
Neurologic			
Developmental delay/intellectual disability	+++ Moderate to severe	+++ Moderate to severe	+++ Mild to severe
Epilepsy/EEG anomalies	++	+	++
Neurobehavioral/psychiatric manifestations	++	+	++
Brain anomalies	++	++	++
Facial features			
Long/triangular face	+++	−	+++
Prominent forehead	+++	+++	+++
Proptosis	−	+++	−
Downslanting palpebral fissures	+++	+++	+++
Underdeveloped midface	−	+++	−
Short nose	++	+++	−
Anteverted nares	++	+++	−
Everted lips	++	++	+
Small chin	−	+++	−
Prominent chin	+++	−	+++
Ophthalmological features			
Vision impairments	+++	++	+
Blue sclerae	++	+++	−
Airway obstructions	−	+++	−
Musculoskeletal			
Advanced bone age	+++	+++	+++
Slender habitus	+++	−	++
Scoliosis	+++	++	++
Pectus carinatum/excavatum	++	+	+
Cardiovascular anomalies	−∼++^∗^	+	+∼++
Other			
Hypertrichosis	−	++	−
Gum hypertrophy	−	++	−

*Note:* This table has been compiled from the following sources: Priolo M. 2024 [2]; Macchiaiolo M, et al. 2022 [5]; Tatton‐Brown K, et al. [6]. +++75–100%; ++ 25–75%; + 5–25%; −0–5%.

^∗^Low‐grade mitral regurgitation is the most common finding (31%), but other CHD and aortic root dilatation are less common (4%).

Previous reports have documented cardiovascular disease in Malan syndrome, including dilatation of the great vessels. One case involved pulmonary artery dilatation, and five cases involved aortic dilatation [2–4]. In one case, aortic dilatation progressed between the ages of 35 and 38 years and was associated with dissection [4]. Macchiaiolo et al. evaluated 16 children using echocardiography and found mitral regurgitation in 31% of cases [5]. Huynh et al. reported three cases of aortic dilatation (11%) in a study of adults with Malan syndrome diagnosed at various ages (at birth, 10 years, and 30 years) [7]. In this case, pulmonary artery dilatation was observed in the absence of any hemodynamic abnormalities, such as right ventricular hypertrophy, tricuspid regurgitation, or pulmonary hypertension. The role of the transcription factor NFIX is becoming clearer, particularly in the development of the brain, skeletal muscle, and hematopoietic system [8, 9]. However, the pathophysiological role of NFIX in the cardiovascular system remains unclear. Malan syndrome is characterized by symptoms reminiscent of Marfan syndrome, such as thin body habitus, long fingers, scoliosis, and chest deformities, including pectus carinatum and excavatum. To our knowledge, no association has been reported between the *NFIX* gene and connective tissue disorder‐related genes, including *FBN1*. Furthermore, there are no known gene pathways or associating genes of *NFIX* that are related to cardiovascular development or vascular pathology. It is possible that the phenotype is influenced by genetic and/or environmental modifiers of aortopathy. Further research is necessary to better define the cardiovascular phenotype in patients with *NFIX* gene variants. The clinical significance and progression of great vessel dilatation remain unclear, but cardiac evaluation is important for all individuals with Malan syndrome at the time of diagnosis and during follow‐up.

Pathogenic heterozygous variants in the *NFIX* gene have been identified in individuals with Marshall–Smith syndrome (MSS) [1, 10]. MSS variants cluster in Exons 6 to 10, resulting in a dominant‐negative effect. In contrast, Malan syndrome variants cluster mostly in Exon 2, resulting in haploinsufficiency. One case of aortic root dilatation has been reported in MSS patients [11]. Although individuals with some phenotypic overlaps do exist, Malan syndrome and MSS are two separate clinical entities (Table 1). MSS can be distinguished from Malan syndrome by the absence of macrocephaly and the presence of proptosis, an underdeveloped midface, and micrognathia (Table 1) [2]. In this case, the detected variant was a frameshift variant in Exon 2, resulting in haploinsufficiency. The presence of macrocephaly and the absence of proptosis and micrognathia led to a diagnosis of Malan syndrome. While Malan syndrome is not currently considered a cause of cardiovascular disease or cardiac abnormalities, further research on cases involving aortic and pulmonary artery dilatation is warranted.

## Author Contributions

Toshihiko Mori wrote the manuscript and provided medical care for the patient. Mayu Hirano also provided medical care for the patient. Shigeto Fuse performed the cardiovascular examination. Maki Katai performed the ophthalmological examination. Hiroko Shigetomi assisted with the diagnosis using AI facial recognition software (Face2Gene) and interpreted the gene analysis results.

## Funding

No funding was received for this manuscript.

## Disclosure

All authors read and approved the final manuscript.

## Ethics Statement

We obtained written informed consent from the patient’s parents for the genetic analysis. We also obtained written informed consent from the patient’s parents to report this case and publish its associated photographs.

## Consent

Please see the Ethics Statement.

## Conflicts of Interest

The authors declare no conflicts of interest.

## Data Availability

The data that support the findings of this study are available on request from the corresponding author. The data are not publicly available due to privacy or ethical restrictions.
